# Continuing efficacy of milnacipran following long-term treatment in fibromyalgia: a randomized trial

**DOI:** 10.1186/ar4268

**Published:** 2013-08-16

**Authors:** Daniel J Clauw, Philip J Mease, Robert H Palmer, Joel M Trugman, Yong Wang

**Affiliations:** 1 University of Michigan Medical School, 24 Frank Lloyd Wright Drive, PO Box 385, Ann Arbor, MI 48106; 2 Swedish Medical Center and the University of Washington School of Medicine, 1101 Madison Street, Seattle, WA 98104; 3 Forest Research Institute, Harborside Financial Center, Plaza V, Suite 1900, Jersey City, NJ 07311

## Abstract

**Introduction:**

Previous studies of long-term treatment response in fibromyalgia and other chronic pain states have generally been limited to approximately one year, leaving questions about the longer-term durability of response. The purpose of this study was to demonstrate continuing efficacy of milnacipran by characterizing changes in pain and other fibromyalgia symptoms after discontinuing long-term treatment. The mean length of milnacipran treatment at the time of randomized withdrawal was 36.1 months from initial exposure to milnacipran (range, 17.9 to 54.4 months).

**Methods:**

After completing a long-term, open-label, lead-in study of milnacipran (which followed varying periods of exposure in previous studies), adult patients with fibromyalgia entered the four-week open-label period of the current study for evaluation of ongoing treatment response. After the four-week period to confirm new baseline status, 151 patients taking milnacipran ≥100 mg/day and reporting ≥50% improvement from pre-milnacipran exposure in Visual Analogue Scale (VAS) pain scores were classified as responders. These responders entered the 12-week, double-blind withdrawal period in which they were randomized 2:1 to continue milnacipran or switched to placebo. The prespecified primary parameter was loss of therapeutic response (LTR), defined as increase in VAS pain score to <30% reduction from pre-milnacipran exposure or worsening of fibromyalgia requiring alternative treatment. Adverse events and vital signs were also monitored.

**Results:**

Time to LTR was shorter in patients randomized to placebo than in patients continuing milnacipran (*P *< 0.001). Median time to LTR was 56 days with placebo and was not calculable for milnacipran, because less than half of the latter group of patients lost therapeutic response by study end. Additionally, 81% of patients continuing on milnacipran maintained clinically meaningful pain response (≥30% improvement from pre-milnacipran exposure), compared with 58% of patients switched to placebo (sensitivity analysis II; *P *< 0.001). The incidences of treatment-emergent adverse events were 58% and 47% for placebo and milnacipran, respectively. Mean decreases in blood pressure and heart rate were found in both groups, with greater decreases for patients switched to placebo.

**Conclusions:**

Continuing efficacy of milnacipran was demonstrated by the loss of effect following withdrawal of treatment in patients who received an average of three years of milnacipran treatment.

**Trial registration:**

ClinicalTrials.gov: NCT01014585

## Introduction

Fibromyalgia is a complex disorder characterized by chronic widespread pain, fatigue, stiffness, poor sleep, depressed mood and cognitive difficulties [1-3]. On the basis of the 1990 American College of Rheumatology (ACR) classification criteria, which focus on pain and tenderness [4], it has been estimated that fibromyalgia affects 2% to 4% of the general population [5,6]. However, in a recent population-based study that used modified ACR 2010 diagnostic criteria [7], which incorporate many of the other hallmark symptoms of fibromyalgia [8], the estimated prevalence was 6.4%. Because ongoing therapy is often required to manage the chronic symptoms of fibromyalgia, demonstration of a medicine's long-term efficacy is of great interest to both clinicians and patients.

Centrally acting analgesics, such as the tricyclic compounds, were investigated once it became clear that the diffuse pain and other symptoms of fibromyalgia were likely due to aberrations in the central nervous system. Preliminary studies with amitriptyline and cyclobenzaprine showed efficacy in patients receiving one to three months of treatment [9,10]. These initially promising results were not replicated in subsequent longer-term studies, and tolerability is always an issue when using tricyclic compounds. Tramadol, another centrally acting analgesic that likely acts by largely augmenting serotonergic and noradrenergic activity (along with some weak μ-opioid effects) has also been shown to have short-term efficacy in patients with fibromyalgia [11-13].

As the pathophysiology of fibromyalgia became better understood, research focused on other centrally acting analgesics that would likely improve this condition [14]. This led to the approval of three medications by the US Food and Drug Administration (FDA) for the management of fibromyalgia: pregabalin, duloxetine and milnacipran. FDA approval of these drugs was based on results from large, randomized, placebo-controlled trials ranging from three to six months in duration [15-17]. In addition to each compound demonstrating efficacy in at least two of these pivotal trials, all three compounds were evaluated in extension studies that ranged from three months to one year in duration [18-21]. The longest fibromyalgia study to date was an open-label trial in which patients received up to 3.25 years of milnacipran after having participated in prior placebo-controlled trials or extension studies [22].

Although treatment benefits were found in the extension studies and the open-label milnacipran study, none included a placebo control group, because placebo-controlled, parallel-group clinical trials are ethically and practically difficult to conduct over long periods of time in patients with chronic pain. Therefore, this randomized withdrawal study was conducted to investigate the effects of discontinuing treatment of fibromyalgia patients who had completed participation in the long-term, open-label milnacipran study [22]. This design provided the opportunity to use a randomized, double-blind, placebo-controlled method to evaluate the long-term efficacy of milnacipran in patients with fibromyalgia.

## Materials and methods

### Design overview

Enrollment began on 20 November 2009, and the last patient visit was completed on 7 June 2010. The study was conducted at 58 US centers and approved by the institutional review board or ethics committee at each site (Appendix 1, Additional File 1), and complied with Good Clinical Practice guidelines and the Declaration of Helsinki. All patients provided their written informed consent to participate in the study.

### Participants

Eligible patients were adults meeting the 1990 ACR criteria for fibromyalgia [4] who entered directly from a long-term, open-label, flexible-dose, lead-in study in which they received milnacipran 50 mg/day to 200 mg/day for up to 3.25 years [22]. Prior to this lead-in study, patients had received up to 15 months of treatment with milnacipran 100 mg/day or 200 mg/day during double-blind studies [20,21,23-27], resulting in up to 4.5 years of milnacipran exposure prior to entering into the current discontinuation study.

Key exclusion criteria included significant risk of suicide, history of serious psychiatric disorder, substantial alcohol use or abuse, pregnancy or breastfeeding, cardiovascular disease within the past 12 months, mean systolic blood pressure >180 mmHg or diastolic blood pressure >110 mm Hg, uncontrolled narrow-angle glaucoma, active liver disease, severe renal impairment and any other medical disorder that might preclude participation as judged by the principal investigator. Certain pharmacologic agents were prohibited during the study, including monoamine oxidase inhibitors, stimulant medications, anorectic agents, daily opiates, sodium oxybate and anesthetic and/or opiate patches. Although daily opiates were prohibited, intermittent use was allowed as needed, except during the seven days before scheduled study visits.

### Randomization and interventions

The study included four weeks during which the patients receiving the same milnacipran dosage as in the lead-in study (25 mg to 100 mg twice daily), twelve weeks with patients randomized to milnacipran or placebo (double-blind) and one week of tapering (double-blind). After the four-week open-label period, patients receiving a minimum dosage of milnacipran 100 mg/day and achieving ≥50% pain improvement from pre-milnacipran exposure (that is, baseline Visual Analogue Scale (VAS) pain score prior to any milnacipran treatment in any study) were classified as responders and randomized (2:1) to continue milnacipran at the same dosage or were switched to placebo. Pain improvement was assessed using 24-hour recall VAS pain scores (range, 0 = "no pain" to 100 = "worst possible pain"). The 50% threshold was not selected as a benchmark for clinically meaningful pain reduction, but rather to enrich the population of responders so that loss of response after randomization (that is, <30% pain reduction from pre-milnacipran exposure and/or worsening of fibromyalgia requiring alternative treatment) could be measured. Patients not meeting the protocol-defined responder criteria were also randomized and followed in the study. However, because these patients were not the primary focus of the study, their results are not presented in this report.

Randomization codes were generated and securely stored by Forest Research Institute, Inc (Jersey City, NJ, USA). An interactive voice and/or web response system was used to calculate the mean pain intensity score during the last week of the open-label period, to allocate patients to the appropriate responder or nonresponder population and to dispense investigational medications, which were sealed and coded to maintain the double-blinding.

### Assessments

The primary efficacy parameter was time to loss of therapeutic response (LTR), defined as time from baseline (randomization) to the first double-blind study visit in which a patient had <30% reduction in VAS pain from pre-milnacipran exposure or worsening of fibromyalgia requiring alternative treatment, as judged by the study's principal investigator. The 30% threshold was based on an accepted definition of clinically meaningful pain reduction [28]. Patients completed 24-hour recall VAS pain assessments for seven days before each study visit. Pain intensity was calculated as the weekly average of daily assessments. Three sensitivity analyses were performed to confirm findings from the primary analysis using the following LTR definitions: (1) <30% reduction in VAS pain score from pre-milnacipran exposure or withdrawal from the study for any reason, (2) <30% reduction in VAS pain score from pre-milnacipran exposure and (3) worsening of fibromyalgia requiring alternative treatment. The second sensitivity analysis was used to determine the percentage of patients who maintained clinically meaningful pain improvements from pre-milnacipran exposure (≥30% reduction in VAS pain score).

Secondary efficacy parameters included time to worsening based on the Patient Global Impression of Change (PGIC) and the Multidimensional Assessment of Fatigue (MAF) global fatigue index [29]. Based on a seven-point scale, worsening in PGIC was defined as patients' reporting their overall change in fibromyalgia from randomization as "much worse" or "very much worse" (score 6 or 7). Worsening in MAF was defined as a 10-point increase from randomization in the fatigue global index score (1 = "no fatigue" to 50 = "severe fatigue").

Additional efficacy parameters included time to worsening in Short Form-36 Health Survey (SF-36) Physical Component Summary (PCS) and Mental Component Summary (MCS) scores [30], with worsening defined as a six-point decrease from randomization. Mean score changes from randomization in VAS pain, Brief Pain Inventory (BPI) [31], Revised Fibromyalgia Impact Questionnaire (FIQR) [32], SF-36 PCS and MCS, and Multiple Ability Self-Report Questionnaire (MASQ) [33] were also analyzed.

Adverse events, vital signs and clinical laboratory tests were monitored for safety. Treatment emergent adverse events were defined as adverse events that occurred after the first dose of double-blind treatment or increase in severity during this period. Efficacy and safety assessments were conducted at all study visits with the exception of PGIC, which was assessed at weeks 2, 4, 8 and 12 of the double-blind period.

### Statistical analyses

Assuming that 60% of the placebo and 35% of the milnacipran groups would experience LTR by end of the double-blind period, it was estimated that 180 enrolled patients (placebo = 60 and milnacipran = 120) would be needed to detect a 25% difference between treatment groups with 90% power at the two-sided 5% significance test. The safety population included all randomized patients who received one or more doses of double-blind treatment. The intent-to-treat population included patients in the safety population who had one or more postrandomization pain assessments. Kaplan-Meier estimates were used to analyze time to LTR and time to worsening based on PGIC, MAF, SF-36 PCS and SF-36 MCS scores, with comparisons between treatment groups analyzed using the logrank test. Hazard ratios (HRs) with 95% confidence intervals (95% CIs) were calculated using a Cox proportional hazards model with treatment group as the explanatory variable. Patients who completed the study without experiencing LTR or who withdrew for any reason other than LTR were censored in the primary efficacy analyses.

An analysis of covariance model was used to calculate mean changes from randomization, with treatment group and study center as factors and baseline value as a covariate. Missing data were imputed using the last observation carried forward approach. Tests were two-sided performed at the 5% level of significance. Descriptive statistics were used to analyze changes from randomization in vital signs and clinical laboratory values, with the end of the study defined as the last available assessment in the double-blind period. All analyses were performed using SAS version 9.1.3 software (SAS Institute, Cary, NC, USA).

## Results

### Patients

**Figure 1:**
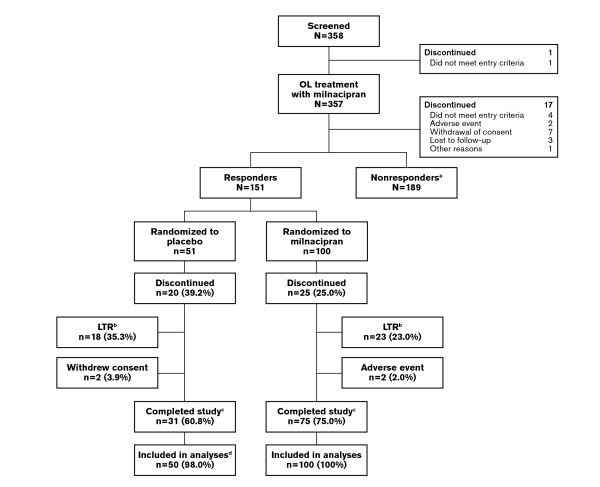
**Study flow**. Responders were defined as patients who received a minimum dosage of milnacipran 100 mg/day and achieved ≥50% pain improvement after long-term treatment. ^a^Patients not meeting responder criteria were analyzed separately, but the results for these patients are not presented in the current report. "Nonresponder" does not necessarily imply no improvement. ^b^Worsening of fibromyalgia requiring alternative treatment, which was one of the loss of therapeutic response (LTR) criteria used for the primary efficacy analysis and for sensitivity analysis III. ^c^Patients reaching the final study visit. ^d^One patient who did not receive at least one dose of the study drug was excluded from safety and efficacy analyses. OL = open-label.

Of the 340 patients who completed the four-week open-label period, 151 (44.4%) were classified as responders and randomized to continue milnacipran (*n *= 100) or switched to placebo (*n *= 51) (Figure 1). Of the patients randomized to continue milnacipran, 75 patients (75.0%) completed the study and 31 patients (60.8%) randomized to placebo completed the study. Most discontinuations during the double-blind period were due to worsening of fibromyalgia requiring alternative treatment, which was one of the LTR definitions used in this study. Two patients randomized to milnacipran discontinued due to an adverse event. One patient randomized to placebo did not receive double-blind treatment and was therefore excluded from safety and efficacy analyses.

Among the 150 patients analyzed for safety and efficacy, demographics and clinical characteristics were similar between treatment groups at randomization (Table 1). In these patients, mean total exposure prior to randomization (that is, total duration of milnacipran treatment in all prior studies and the four-week open-label period of this study) was 36.1 months (range, 17.9 to 54.4 months). Total duration of milnacipran exposure prior to randomization was as follows (percentage of patients): more than 12 to 24 months (14%), more than 24 to 36 months (39%), more than 36 to 48 months (33%) and more than 48 to 60 months (14%).

**Table 1:** Patient demographics and baseline characteristics^a^

Demographics and characteristics	Placebo (*n *= 50)	Milnacipran (*n *= 100)	Total (*N *= 150)
Mean age (SD), years	54.0 (8.3)	54.5 (9.3)	54.3 (9.0)
Women, *n *(%)	48 (96.0)	96 (96.0)	144 (96.0)
Race, *n *(%)			
White	47 (94.0)	96 (96.0)	143 (95.3)
Nonwhite	3 (6.0)	4 (4.0)	7 (4.7)
Mean weight (SD), kg	77.3 (16.7)	80.2 (14.5)	79.2 (15.3)
Mean body mass index (SD), kg/m^2^	29.0 (6.1)	29.7 (5.2)	29.5 (5.5)
Mean SF-36 PCS score (SD)	41.3 (10.2)	41.6 (8.4)	41.5 (9.0)
Mean SF-36 MCS score (SD)	53.6 (11.3)	53.6 (9.0)	53.6 (9.8)
Mean FIQR total score (SD), range 0 to 100	21.4 (15.8)	19.4 (11.9)	20.1 (13.3)
Mean MAF global fatigue score (SD), range 1 to 50	21.4 (10.4)	20.7 (9.7)	21.0 (9.9)
Mean BPI average pain score (SD), range 0 to 10	2.5 (1.3)	2.3 (1.4)	2.3 (1.4)
Mean VAS pain score (SD), range 0 to 100	19.3 (11.6)	16.6 (9.6)	17.5 (10.3)
Mean VAS pain score (SD), pre-milnacipran exposure^b^	66.2 (14.7)	65.4 (13.0)	65.7 (13.6)

^a^*Baseline *is defined as the randomization visit in this study. BPI = Brief Pain Inventory; FIQR = Revised Fibromyalgia Impact Questionnaire; MAF = Multidimensional Assessment of Fatigue; MCS = Mental Component Summary; PCS = Physical Component Summary; SF-36 = Short Form-36 Health Survey; VAS = Visual Analogue Scale. ^b^Baseline value from lead-in study prior to first milnacipran exposure.

### Efficacy parameters

**Figure 2:**
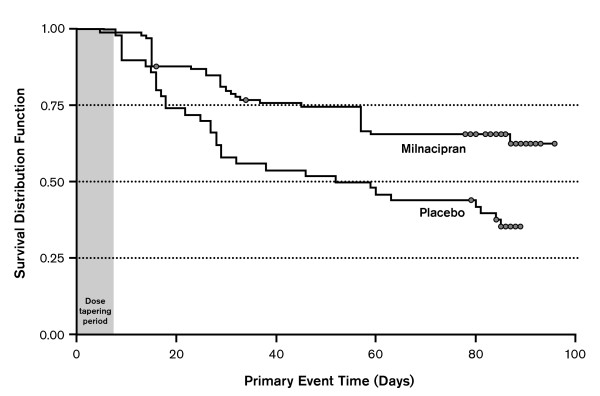
**Kaplan-Meier plot of time to loss of therapeutic response**. Loss of therapeutic response was defined as <30% reduction in Visual Analogue Scale pain score from pre-milnacipran exposure or a worsening of fibromyalgia requiring an alternative treatment. Circles represent censored patients.

Patients discontinuing milnacipran had significantly shorter time to LTR than patients who continued treatment (primary analysis; HR, 0.44 [95% CI, 0.27 to 0.71]; *P *< 0.001) (Figure 2 and Table 2). Median time to LTR was 56 days for placebo and was not calculable for milnacipran, because 50% of patients in the latter group did not experience LTR by the end of the study. At the end of the study, 64% of the patients switched to placebo experienced an LTR compared with 35% of patients who continued milnacipran. Very similar results were found when LTR was defined as more than 30% reduction in VAS pain score from pre-milnacipran exposure or withdrawal from the study for any reason (instead of worsening of fibromyalgia requiring alternative treatment) (sensitivity analysis I; HR, 0.46 [95% CI, 0.29 to 0.74]; *P *= 0.001). In addition, the percentage of patients maintaining 30% or more pain improvement from pre-milnacipran exposure was 81% in the milnacipran group and 58% in the placebo group (sensitivity analysis II; HR, 0.35 [95% CI, 0.19 to 0.65]; *P *< 0.001). The only nonsignificant finding was for sensitivity analysis III, which simply defined LTR as worsening of fibromyalgia requiring alternative treatment.

**Table 2:** Time to loss of therapeutic response^a^

Measurement	Treatment group, *n*	Time to LTR, days (95% CI)^b^		Hazard ratio (95% CI)^c^	*P*-value^d^	Patients with LTR at end of DB treatment, *n *(%)
					
		25th percentile	50th percentile			
Primary analysis	PBO, 50	18 (16 to 29)	56 (28 to 85)	0.44 (0.27 to 0.71)	<0.001	32 (64)
	MLN, 100	45 (29 to 59)	NC			35 (35)
Sensitivity analysis I	PBO, 50	18 (16 to 29)	56 (28 to 85)	0.46 (0.29 to 0.74)	0.001	32 (64)
	MLN, 100	36 (29 to 57)	NC			37 (37)
Sensitivity analysis II	PBO, 50	28 (18 to 63)	85 (60 to NL)	0.35 (0.19 to 0.65)	<0.001	21 (42)
	MLN, 100	NC	NC			19 (19)
Sensitivity analysis III	PBO, 50	29 (17 to NL)	NC	0.59 (0.32 to 1.08)	0.08	18 (36)
	MLN, 100	86 (43 to NL)	NC			24 (24)
PGIC	PBO, 50	22 (15 to 31)	86 (30 to NL)	0.36 (0.20 to 0.63)	<0.001	25 (50)
	MLN, 100	NC	NC			22 (22)
MAF global fatigue	PBO, 50	18 (16 to 68)	NC	0.80 (0.46 to 1.38)	0.41	20 (40)
	MLN, 100	30 (26 to 59)	NC			36 (36)
SF-36 PCS	PBO, 50	15 (15 to 22)	68 (18 to NL)	0.81 (0.50 to 1.30)	0.36	26 (52)
	MLN, 100	23 (15 to 33)	87 (57 to NL)			47 (47)
SF-36 MCS	PBO, 50	28 (16 to 46)	57 (29 to NL)	0.74 (0.44 to 1.24)	0.24	23 (46)
	MLN, 100	29 (23 to 60)	90 (85 to NL)			40 (40)

^a^*Loss of therapeutic response *was defined as follows: (1) primary analysis: <30% reduction in Visual Analogue Scale (VAS) pain score from pre-milnacipran exposure or worsening of fibromyalgia requiring alternative treatment; (2) sensitivity analysis I: <30% reduction in VAS pain score from pre-milnacipran exposure or withdrawal from the study for any reason; (3) sensitivity analysis II: <30% reduction in VAS pain score from pre-milnacipran exposure; or (4) sensitivity analysis III: worsening of fibromyalgia requiring alternative treatment. DB = double-blind; LTR = loss of therapeutic response; MAF = Multidimensional Assessment of Fatigue; MCS = Mental Component Summary; MLN = milnacipran; NC = not calculable (no patients in quartile); NL = no limit; PBO = placebo; PCS = Physical Component Summary; PGIC = Patient Global Impression of Change; SF-36 = Short Form-36 Health Survey. ^b^Quartiles (that is, 25th percentile, 50th percentile) based on Kaplan-Meier estimates. ^c^Hazard ratio of milnacipran versus placebo based on Cox proportional hazards regression model with treatment group as an explanatory variable. ^d^*P*-values based on logrank comparison between milnacipran and placebo.

**Figure 3:**
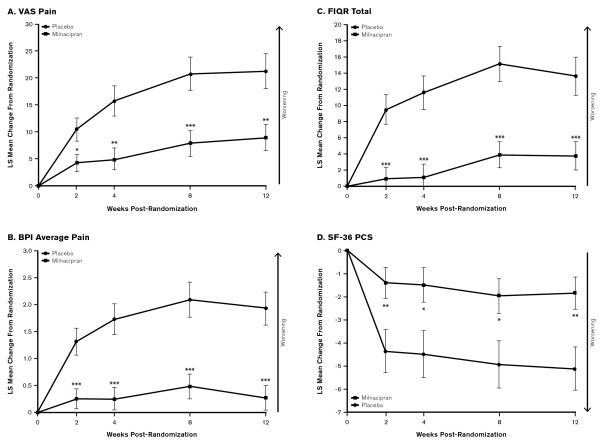
**Least squares mean changes from baseline in additional efficacy outcomes**. Least squares mean changes and standard errors from randomization at weeks 2, 4, 8 and 12 of double-blind treatment (visits 4, 5, 6 and 7), with the last observation carried forward. BPI = Brief Pain Inventory; FIQR = Revised Fibromyalgia Impact Questionnaire; LS = least squares; SF-36 = Short Form-36 Health Survey; VAS = Visual Analogue Scale. *P < 0.05; **P < 0.01; ***P < 0.001 versus placebo.

Patients discontinuing milnacipran had a shorter time to worsening than patients continuing treatment in PGIC, MAF global fatigue, SF-36 PCS and SF-36 MCS scores (Table 2), with significant between-group differences for PGIC (HR, 0.36 [95% CI, 0.20 to 0.63]; *P *< 0.001). In addition, mean least squares changes from randomization indicated greater worsening in VAS pain, BPI average pain, FIQR and SF-36 PCS scores with placebo versus milnacipran, with significant between-group differences observed at all study visits (*P *< 0.05) (Figure 3). Patients switched to placebo also had greater worsening from randomization in mental functioning (SF-36 MCS: placebo, -4.64; milnacipran, -2.79) and cognitive difficulties (MASQ total: placebo, +3.43; milnacipran, +2.45), although differences between treatment groups were not significant.

### Tolerability and safety outcomes

Among the 357 patients receiving milnacipran during the four-week open-label period, treatment emergent adverse events occurring in ≥2.5% of patients were upper respiratory tract infection (3.4%), nasopharyngitis (2.5%) and nausea (2.5%). No serious adverse events occurred during this period, and two patients discontinued due to adverse events (one with nausea and one with hypertension). During the double-blind period, 47% of patients continuing milnacipran and 58% of patients switched to placebo reported one or more treatment emergent adverse events (Table 3). Adverse events led to premature discontinuation in two patients receiving milnacipran (one with nausea and vomiting and one with increased heart rate and hypertension) and no patients receiving placebo. The only serious adverse event (noncardiac chest pain) was reported in one milnacipran-treated patient. No deaths occurred during the study.

**Table 3:** Incidence of treatment-emergent adverse events^a^

Incidence, *n *(%)	Placebo (*n *= 50)	Milnacipran (*n *= 100)
Patients with ≥1 TEAE	29 (58)	47 (47)
Nausea	1 (2)	4 (4)
Headache	0	4 (4)
Vomiting	1 (2)	4 (4)
Sinusitis	3 (6)	4 (4)
Upper respiratory tract infection	2 (4)	4 (4)
Fatigue	1 (2)	4 (4)
Pain in extremity	1 (2)	4 (4)
Fall	0	4 (4)
Arthralgia	1 (2)	3 (3)
Fibromyalgia	1 (2)	3 (3)
Peripheral edema	3 (6)	2 (2)
Hypothyroidism	2 (4)	1 (1)
Influenza	2 (4)	1 (1)
Irritability	3 (6)	0
Neck pain	2 (4)	0

^a^Reported in ≥3% of patients in either treatment group during the double-blind period. TEAE = treatment emergent adverse event.

Mean decreases in sitting vital signs were found in both treatment groups during the double-blind period, with greater decreases observed in patients receiving placebo. Mean changes from randomization to the end of the study were as follows: systolic blood pressure (placebo, -1.3 mmHg; milnacipran, -0.7 mmHg), diastolic blood pressure (placebo, -3.4 mmHg; milnacipran, -0.8 mmHg) and pulse (placebo, -10.6 beats/min; milnacipran, -2.7 beats/min). Potentially clinically significant changes in vital signs were found in one patient receiving milnacipran (heart rate, ≥120 beats/min with increase ≥20 beats/min from randomization) and one patient receiving placebo (heart rate, ≤50 beats/min with decrease of ≥15 beats/min from randomization). No potentially clinically significant change in laboratory parameters occurred in more than two patients in either treatment group.

## Discussion

Because it can be difficult to conduct long-term, placebo-controlled, parallel-arm studies to assess the efficacy of chronic pain medications, we used a randomized, placebo-controlled, withdrawal design in this trial to evaluate the loss of therapeutic response in patients with fibromyalgia who discontinued long-term treatment with milnacipran. As in other withdrawal studies [34], including the **F**uture **Re**vascularization **E**valuation in patients with Diabetes Mellitus: **O**ptimal Management of **M**ultivessel Disease (**FREEDOM**) study of pregabalin in fibromyalgia [35], the present study focused on a subset of patients who responded to treatment. Although there are differences in design between the present study and FREEDOM, and though it would be improper to make any formal comparison between the two studies, it is of interest that both showed a loss of response in almost two-thirds of patients who were taken off therapy. It is also true that both studies showed a loss of response in almost one-third of patients continuing on therapy-attesting, perhaps, to the underlying variability in the pain of fibromyalgia. The fact that approximately one-third of patients switched to placebo in each study did not lose their therapeutic response is also of interest. Again, this may reflect the natural variability in the disease; in either case, there is sufficient evidence to suggest that these medications had a pharmacologic effect on the fundamental disturbances in pain processing that are characteristic of fibromyalgia [36].

In the current study, we analyzed data from patients who achieved ≥50% pain improvement after receiving 1.5 to 4.5 years (mean, 3 years) of milnacipran treatment in one or more prior clinical trials. Milnacipran dosages had been optimized for each patient entering the withdrawal study (50 mg/day to 200 mg/day), although the present analyses were limited to patients receiving at least the minimum recommended dosage of 100 mg/day [17]. The results of these analyses indicate that the patients classified as responders had experienced clinical benefits during long-term treatment with milnacipran, as evidenced by a shorter time to LTR and greater worsening in symptom severity postrandomization among the patients who were switched to placebo (that is, discontinued treatment) as compared to those who continued milnacipran. For the prespecified primary endpoint, significantly more patients in the placebo group experienced an LTR by the end of study compared to the milnacipran group (64% vs. 35%, *P *< 0.001).

The primary endpoint, as well as two of the three sensitivity analyses, included either worsening of fibromyalgia or discontinuation from the study as a definition of LTR. Sensitivity analysis II, however, was based solely on the pain criterion (that is, <30% reduction in VAS pain score from pre-milnacipran exposure). The results of this analysis, in conjunction with analyses of mean pain scores at various time points (that is, pre-milnacipran exposure, study randomization and end of study), may provide some important insights about pain responses in patients who had received up to 4.5 years of prior milnacipran treatment. First, notable pain improvements were found in patients who received long-term milnacipran treatment, as indicated by the 73% decrease in mean VAS pain score from pre-milnacipran exposure (65.7%) to randomization (17.5%) (Table 1). Of course, this result was derived from a selected group of responders who elected to continue treatment for an extended period of time, and the large mean decrease in pain does not necessarily imply that long-term treatment increases response to milnacipran. Nonetheless, the results from sensitivity analysis II indicate that, after randomization, the percentage of patients maintaining clinically meaningful pain improvement (that is, >30% decrease in pain from pre-milnacipran exposure) was higher in the group continuing milnacipran (81%) than in the group switched to placebo (58%) (Table 2). Although mean increases in VAS and BPI pain scores from randomization to the end of the study were observed in both treatment groups, worsening was greater in patients who discontinued milnacipran. There is no definitive explanation for the slight increase in mean pain scores in patients continuing milnacipran, but because randomization was based on achieving a certain level of pain reduction (≥50%), it is likely that some regression to the mean in pain severity occurred in this group. It is also possible that the results reflect some patients' assumptions that they had been discontinued from active treatment (for example, a "reverse placebo" or nocebo effect) [37].

This study also demonstrated that improvements in global status (PGIC), fibromyalgia severity (FIQR) and physical functioning (SF-36 PCS) occurred in patients receiving long-term milnacipran treatment. A higher percentage of patients discontinuing milnacipran reported feeling "much worse" or "very much worse" since randomization as compared with patients who continued treatment (50% vs. 22%, *P *< 0.001). These findings were supported by mean worsening from randomization in FIQR and SF-36 PCS scores, which were significantly worse with placebo than milnacipran at every study visit.

In patients who discontinued treatment, the greatest worsening in mean SF-36 PCS scores occurred between randomization and week 2, whereas mean VAS pain scores worsened steadily from randomization to week 8 (Figure 3). These results are consistent with findings in an earlier milnacipran study that included 12 weeks of double-blind treatment followed by a 2-week placebo-controlled discontinuation period [38]. The results from the short discontinuation period in that study also seem to indicate that physical functioning worsens more rapidly than pain after withdrawal of treatment. The reasons for these findings are unknown, but one possible explanation is that the function measures used in previous milnacipran studies were more sensitive than pain measures for detecting loss of response. It is also possible that when fibromyalgia patients experience a decline in physical function after discontinuing treatment, they decrease their activity levels, which in turn prevents them from experiencing increased pain severity. Alternatively, the results may simply indicate that the effects of milnacipran on pain are more persistent than its effects on functioning.

In addition to demonstrating the continuing efficacy of milnacipran, the worsening of pain and other symptoms observed in this withdrawal study confirms the benefit of ongoing treatment in patients with fibromyalgia. Moreover, these results suggest that the underlying pathophysiology of fibromyalgia was been reversed by long-term treatment, which has important implications for our understanding of this disorder-namely, that even a reasonably successful treatment, as demonstrated in this study by the ≥50% pain improvement from pre-milnacipran exposure at randomization, is not sufficient to "cure" individuals with this disorder by somehow resetting pain processing. This finding, combined with the fact that spontaneous remission is very uncommon, suggests that individuals with fibromyalgia who experience significant benefit with initial therapy (at least with this compound) should expect to continue to require long-term treatment.

No unexpected safety issues were observed in this study. Nausea and headache, the most commonly reported treatment emergent adverse events with milnacipran in placebo-controlled trials [25,26,39] and the long-term lead-in study [22], were each observed in 4% of patients continuing milnacipran in this study. In patients discontinuing treatment, the most common treatment emergent adverse events were sinusitis, peripheral edema and irritability (6% each with none rated as severe). Of those symptoms, only irritability is commonly associated with the abrupt discontinuation of serotonin and norepinephrine reuptake inhibitors [16,17,40]. However, patients in this study were not specifically evaluated for a discontinuation syndrome. Mean increases in blood pressure and heart rate have been reported in milnacipran-treated patients during placebo-controlled trials [17]. The results in the present study suggest that the effects of milnacipran on blood pressure and heart rate do not persist after patients discontinue treatment.

A few limitations should be noted. First, on the basis of the results reported above, no conclusions can be drawn about the effects of discontinuing milnacipran in patients who did not meet the stringent ≥50% pain responder criterion. However, subsequent analyses of this study indicate that patients who had 30% to <50% pain improvement from pre-milnacipran exposure, which indicates clinically meaningful improvement, also experienced loss of therapeutic effect after milnacipran was discontinued [41]. Second, although milnacipran dosages were optimized for each patient entering this withdrawal study (50 mg/day to 200 mg/day), the analyses were designed to evaluate only those patients who received at least 100 mg/day, the recommended minimum dosage [17]. Thus, no conclusions can be drawn regarding the effects of any specific dosage. Third, as has been discussed in other randomized withdrawal studies [34,35], some patients may have suspected that they were on placebo if they experienced a clear worsening of symptoms during the 12-week discontinuation period. Any such unblinding effect may have influenced how patients assessed their pain and other symptoms in this study. Nonetheless, significant differences between milnacipran and placebo were observed.

## Conclusions

In patients who received milnacipran for an average of three years (range, 1.5 to 4.5 years), loss of therapeutic response upon discontinuation of treatment provides evidence of long-term efficacy of this medication for the management of fibromyalgia.

## Abbreviations

BPI: Brief Pain Inventory; FIQR: Revised Fibromyalgia Impact Questionnaire; LTR: loss of therapeutic response; MAF: Multidimensional Assessment of Fatigue; MASQ: Multiple Ability Self-Report Questionnaire; MCS: Mental Component Summary; PCS: Physical Component Summary; PGIC: Patient Global Impression of Change; SF-36: Short Form-36 Health Survey; VAS: Visual Analogue Scale.

## Competing interests

DJC has received grants and research support from Pfizer Inc and Forest Laboratories. He has been a consultant for and has served on advisory boards for Pfizer Inc, Eli Lilly and Co, Forest Laboratories, Inc, Cypress Bioscience, Inc (now Royalty Pharma), Pierre Fabre Pharmaceuticals, UCB and AstraZeneca. PJM has received research and grant funding as well as consultation fees from Forest Laboratories, Inc, Cypress Bioscience, Inc, Eli Lilly and Co, Pfizer Inc, Allergan, Inc, Wyeth Pharmaceuticals, Jazz Pharmaceuticals and Fralex Therapeutics. In addition to being full-time employees of Forest Research Institute, Inc, a wholly owned subsidiary of the study sponsor (Forest Laboratories, Inc), RHP, JMT and YW hold stock in the parent company.

## Authors' contributions

DJC and RHP contributed the design of this study. JMT was the study director. PJM was a principal investigator of the study. YW performed statistical analyses of the data. All authors had full access to the data and participated in the interpretation of study results. All authors were also involved in drafting and revising the manuscript. All authors approved the final manuscript for publication.

## Supplementary Material

Additional file 1 **List of Institutional Review Boards and Study Centers**. Click here for file
