# Supplementary material for: Continuing efficacy of milnacipran following long-term treatment in fibromyalgia: a randomized trial
Source: Arthritis Res Ther. 2013 Aug 16;15(4):R88. doi: 10.1186/ar4268 (PMC3978750; doi:10.1186/ar4268)
Supplement: Additional file 1 — List of Institutional Review Boards and Study Centers. [file ar4268-S1.PDF]

APPENDIX 1: List of Institutional Review Boards and Study Centers

Quorum Review IRB (Seattle, WA)

- AAIR Research Center (Rochester, NY)
- Achieve Clinical Research, LLC (Birmingham, AL)
- Advanced Clinical Research—a division of Medford's Women's Clinic, LLC (Medford, OR)
- Altoona Center for Clinical Research (Duncansville, PA)
- Apex Research Institute (Santa Ana, CA)
- Arroyo Medical Group, Inc. (Pismo Beach, CA)
- Arthritis and Osteoporosis Center of Maryland (Frederick, MD)
- Arthritis Clinic (Racine, WI)
- Arthritis Consultants, Inc. (St. Louis, MO)
- Atlanta Research Center, LLC (Atlanta, GA)
- Avancia Clinical Research (Pembroke Pines, FL)
- Catalina Pointe Clinical Research Inc. (Tucson, AZ)
- Central Pennsylvania Clinical Research (Mechanicsburg, PA)
- Clinical Pharmacology Study Group (Worcester, MA)
- Clinical Research Center of CT (Danbury, CT)
- Compass Research, LLC (Orlando, FL)
- Comprehensive NeuroScience, Inc. (Atlanta, GA)
- Connecticut Clinical Research (Cromwell, CT)
- Crescent Medical Research (Salisbury, NC)
- CRC of Jackson (Jackson, MS)
- Deaconess Clinic Downtown Research Institute (Evansville, IN)
- Delray Research Associates (Delray Beach, FL)
- Don L. Goldenberg, MD, PC (Newton, MA)
- East-West Medical Research Institute (Honolulu, HI)
- Fatigue Consultation Clinic (Salt Lake City, UT)
- FutureCare Studies, Inc. (Springfield, MA)
- Infinity Medical Research (North Dartmouth, MA)

- Innovative Clinical Trials (Birmingham, AL)
- Internist Associates of Central New York, PC (Syracuse, NY)
- Knight Center for Integrated Health (Peoria, IL)
- Lake County Research Associates (Libertyville, IL)
- Long Island Clinical Research Associates, LLP (Great Neck, NY)
- Lovelace Scientific Resources, Inc. (Albuquerque, NM)
- Metrolina Medical Research (Charlotte, NC)
- Neuro-Pain Medical Center (Fresno, CA)
- Physicians East, PA (Greenville, NC)
- Piedmont Medical Research (Winston-Salem, NC)
- PRO Research (Eugene, OR)
- Professional Place Medical Group, LLC (Chesapeake, VA)
- Progressive Clinical Research (Vista, CA)
- Radiant Research, Inc. (Anderson, SC)
- Radiant Research, Inc. (Columbus, OH)
- Radiant Research, Inc. (Greer, SC)
- Rapid Medical Research Inc. (Cleveland, OH)
- Renstar Medical Research (Ocala, FL)
- Rheumatology, PC (Kalamazoo, MI)
- San Diego Arthritis Medical Clinic (San Diego, CA)
- Stamford Therapeutics Consortium (Stamford, CT)
- Sunstone Medical Research, LLC (Medford, OR)
- Superior Research LLC (Sacramento, CA)
- The Arthritis Center (Palm Harbor, FL)
- The Portland Clinic, LLC (Portland, OR)
- Vital re:Search (Greensboro, NC)
- Wenatchee Valley Medical Center (Wenatchee, WA)
- Willamette Valley Clinical Studies (Eugene, OR)

Western Institutional Review Board (Olympia, WA)

- Swedish Rheumatology Associates (Seattle, WA)
- UM.D.NJ (Cherry Hill, NJ)

University of Cincinnati, Institutional Review Board (Cincinnati, OH)

- University of Cincinnati – Medical Arts Building (Cincinnati, OH)
